# Neuromagnetic Index of Hemispheric Asymmetry Prognosticating the Outcome of Sudden Hearing Loss

**DOI:** 10.1371/journal.pone.0035055

**Published:** 2012-04-20

**Authors:** Lieber Po-Hung Li, An-Suey Shiao, Kuang-Chao Chen, Po-Lei Lee, David M. Niddam, Shyue-Yih Chang, Jen-Chuen Hsieh

**Affiliations:** 1 Department of Otolaryngology, Cheng Hsin General Hospital, Taipei, Taiwan; 2 Integrated Brain Research Laboratory, Department of Medical Research and Education, Taipei Veterans General Hospital, Taipei, Taiwan; 3 Department of Otolaryngology, Taipei Veterans General Hospital, Taipei, Taiwan; 4 Faculty of Medicine, National Yang-Ming University, Taipei, Taiwan; 5 Brain Research Center, National Yang-Ming University, Taipei, Taiwan; 6 Institute of Brain Science, National Yang-Ming University, Taipei, Taiwan; 7 Department of Electrical Engineering, National Central University, Taoyuan, Taiwan; Hangzhou Normal University, China

## Abstract

The longitudinal relationship between central plastic changes and clinical presentations of peripheral hearing impairment remains unknown. Previously, we reported a unique plastic pattern of “healthy-side dominance” in acute unilateral idiopathic sudden sensorineural hearing loss (ISSNHL). This study aimed to explore whether such hemispheric asymmetry bears any prognostic relevance to ISSNHL along the disease course. Using magnetoencephalography (MEG), inter-hemispheric differences in peak dipole amplitude and latency of N100m to monaural tones were evaluated in 21 controls and 21 ISSNHL patients at two stages: initial and fixed stage (1 month later). Dynamics/Prognostication of hemispheric asymmetry were assessed by the interplay between hearing level/hearing gain and ipsilateral/contralateral ratio (I/C) of N100m latency and amplitude. Healthy-side dominance of N100m amplitude was observed in ISSNHL initially. The pattern changed with disease process. There is a strong correlation between the hearing level at the fixed stage and initial I/C_amplitude_ on affected-ear stimulation in ISSNHL. The optimal cut-off value with the best prognostication effect for the hearing improvement at the fixed stage was an initial I/C_latency_ on affected-ear stimulation of 1.34 (between subgroups of complete and partial recovery) and an initial I/C_latency_ on healthy-ear stimulation of 0.76 (between subgroups of partial and no recovery), respectively. This study suggested that a dynamic process of central auditory plasticity can be induced by peripheral lesions. The hemispheric asymmetry at the initial stage bears an excellent prognostic potential for the treatment outcomes and hearing level at the fixed stage in ISSNHL. Our study demonstrated that such brain signature of central auditory plasticity in terms of both N100m latency and amplitude at defined time can serve as a prognostication predictor for ISSNHL. Further studies are needed to explore the long-term temporal scenario of auditory hemispheric asymmetry and to get better psychoacoustic correlates of pathological hemispheric asymmetry in ISSNHL.

## Introduction

Functional imaging of brain reorganization and neurodynamics in response to central lesions provides essential information related to the prognosis of animals [1], which in turn might assist in the treatment policy for improved functional recovery of human beings. It has been shown that the initial reactions of central auditory pathway after acute injury to the peripheral receptor organ may bear a considerable effect on the final outcome of hearing function in animal studies [2]. However, the contingency between central plastic changes and prognosis along the disease course of a peripheral hearing impairment in human beings remains unexplored.

Idiopathic sudden sensorineural hearing loss (ISSNHL) is a good disease model for the study of the association between auditory neuroplasticity and clinical presentations. ISSNHL, a disease entity of unknown pathogenesis, is widely varied in the presenting signs and prognosis. Though it is possible that the neural deficit(s) lie at a higher level of the auditory pathway, the cochlea has generally been considered the most probable lesion site of ISSNHL. About one third to a half of affected persons achieve partial or complete recovery of hearing after appropriate interventions [3]. The hearing generally reached a fixed level about one month after the treatment [4]. Yet, there is no reliable biomarker that can prognosticate the eventual outcome and/or hearing level in ISSNHL.

By choosing patients with mild-to-moderate hearing impairment, we have previously confirmed by means of MEG that acute unilateral ISSNHL can induce functional reorganization in terms of altered hemispheric asymmetry for sound processing in the central auditory pathway on either affected- or healthy-ear stimulation [5], [6]. In contrast to the pattern of “contralateral dominance” in controls, a pattern of “healthy-side dominance” of N100m to tone burst stimulation was observed in patients. This asymmetry was manifested as stronger dipole moments to monaural acoustic stimuli over hemisphere ipsilateral to healthy ear irrespective on either healthy- or affected-ear stimulation. On affected-ear stimulation, N100m dipole moment was significantly stronger in the contralateral than in the ipsilateral hemisphere (i.e. contralateral dominance). On healthy-ear stimulation, however, N100m dipole moment was significantly stronger in the ipsilateral than in the contralateral hemisphere (i.e. ipsilateral dominance).

Our MEG findings are corroborated by a functional magnetic resonance imaging (fMRI) study in which loss of contralateral dominance on healthy-ear stimulation in patients with unilateral ISSNHL was reported [7]. Functional MRI observations of healthy-side dominance were evidenced by a greater spatial extent (more significant voxels) as activated by auditory stimulation. The abnormal pattern of “ipsilateral dominance” on healthy-ear stimulation in the acute stage of ISSNHL revealed a tendency toward a symmetrical pattern along the recovery course one month later. The report characterizing auditory brain activation in patients with unilateral ISSNHL suggests a dynamic plasticity of central auditory pathways. It has been shown that the pattern of auditory evoked fields (AEFs) of “healthy-side dominance” in the acute stage of unilateral ISSNHL could be evolving in the later course of disease [8]. However, to our knowledge, no study on the prognostic relevance of hemispheric asymmetry coupled to hearing function in ISSNHL has been reported.

We thus investigated whether or not the pattern of hemispheric asymmetry bears any prognostic effect with respect to the recovery of function in patients with acute unilateral ISSNHL by using MEG in this study. AEFs were assessed by measuring N100m in ISSNHL (initial visit and one month after the treatment, respectively) and in normal hearing subjects (once only during the study). Ipsilateral/contralateral ratio was used to assess the degree of hemispheric asymmetry during follow-ups and determine the prognostic relevance to unilateral ISSNHL [9].

## Methods

### Ethics Statement

The study was in compliance with Declaration of Helsinki. Written informed consent was obtained from each participant with a protocol approved by the Institutional Ethics and Research Committee of Taipei Veterans General Hospital and Cheng Hsin General Hospital.

### Subjects (Table 1)

Twenty-one right-handed, previously untreated adult patients with acute unilateral left (n = 11) or right (n = 10) ISSNHL (12 males; 21–70 years of age, mean = 46) were studied. Initial MEG responses of thirteen patients had been reported in our preliminary one-time point studies [5], [6]. Sensorineural hearing loss was diagnosed according to the criteria of a threshold no less than 30 dB HL over three contiguous frequencies within three days or less [10]. No other neurological deficits or traumatic history were identified. All patients received treatments consisting of parenteral steroids and common oral rheological drugs including pentoxifylline and nicametate citrate for five days during the admission. Outpatient therapy with oral rheological drugs ceased by one month for patients with partial and no recovery at discharge. Elapsed time for the initial MEG and pure tone audiometry (PTA) exam after disease onset ranged from 2 days to 3 weeks. MEG and PTA exam were then repeated at about 1 month after initial exam (i.e., fixed stage) [11]. The average threshold of 500 Hz, 1000 Hz, and 2000 Hz according to the last audiogram was exploited to split patients into three prognostic subgroups: complete recovery, partial recovery, and no recovery. Hearing improvement was conventionally defined as: (1) a threshold of>25 dB HL in the affected ear (complete recovery), or (2) a threshold of >25 dB HL with a gain of >10 dB HL in the affected ear (partial recovery) [12], [13].

**Table 1:** General data for all participants.

	Control		ISSNHL patient										
							Th (dB)		De (dB)				
							Initial		Initial		1m		
No	Gender	Age (yr)	Gender	Age (yr)	Du (d)	Lesion	Avg	1 k	Avg	1 k	Avg	1 k	Improvement
1	M*	35	M*	35	17	Rt	12	15	53	50	15	10	c
2	M*	25	M*	70	21	Lt	20	20	60	65	40	50	p
3	M*	29	M*	43	7	Lt	15	10	65	65	10	10	c
4	M*	34	M*	34	8	Rt	15	20	50	60	62	65	n
5	F*	40	M	49	8	Rt	18	20	63	65	63	65	n
6	F*	42	F	56	4	Rt	10	10	47	40	38	35	n
7	F*	46	M*	48	8	Lt	17	20	63	60	62	65	n
8	M	36	F	55	21	Rt	15	20	40	40	35	30	n
9	M	26	F*	50	10	Rt	10	20	63	60	43	35	p
10	M*	66	F	45	4	Rt	18	20	65	65	53	55	p
11	F	26	F*	35	17	Rt	10	15	50	55	50	55	n
12	M	36	F*	53	7	Lt	20	15	63	55	53	45	n
13	M*	25	F	51	10	Lt	8	10	45	50	42	45	n
14	F	26	M	44	2	Lt	18	20	55	50	53	50	n
15	F	27	M*	55	3	Lt	7	10	48	45	18	15	c
16	M	36	M*	21	9	Lt	20	20	62	60	43	45	p
17	F*	62	F*	29	9	Lt	12	15	52	55	42	35	n
18	F*	54	F	53	6	Lt	15	15	65	65	53	60	p
19	M*	21	F*	27	21	Rt	10	5	33	35	28	30	n
20	F*	34	M	41	10	Lt	17	15	40	40	22	20	c
21	M	23	M	70	20	Rt	18	20	52	60	45	55	n

No, participant number; Age, y/o; Du, time elapsed since onset of hearing loss to initial MEG exam (days); Lesion, ear of hearing loss; Lt, left ear; Rt, right ear; Th, initial hearing threshold of the healthy ear (dB HL); De, degree of hearing loss of the affected ear (dB HL); Avg, average hearing threshold of 500 Hz, 1000 Hz, and 2000 Hz; 1 k, hearing threshold at 1000 Hz; Improvement, hearing improvement defined as: (1) a threshold of ≤ 25 dB HL in the affected ear (complete recovery, c), or (2) a threshold of >25 dB HL with a gain of >10 dB HL in the affected ear (partial recovery, p); n, no recovery; Initial, initial PTA exam; 1 m, 1 month after initial exam (fixed stage); *, participants who were involved in our previous studies (see methods).

Twenty-one right-handed healthy volunteers with normal hearing (12 males; 21–66 years of age, mean = 36) served as control. Thirteen controls were involved in our previously published studies [5], [6].

### Audiometric and Electrophysiological Exam

**Figure 1:**
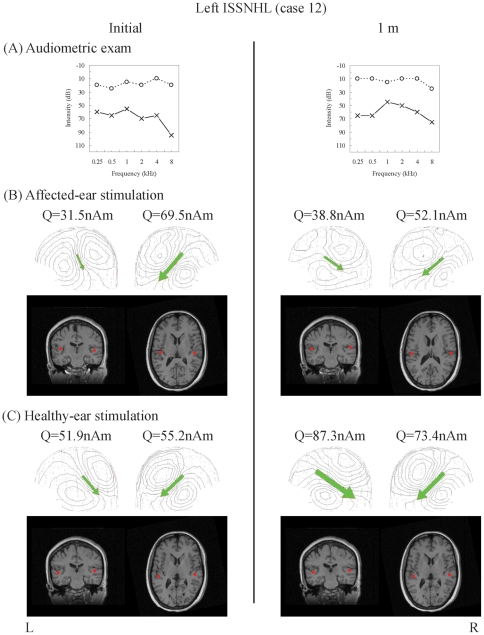
Temporal dynamics of neuromagnetic responses to monaural stimulation with reference to hearing status in unilateral ISSNHL patients. Patient 12 (female, left ISSNHL) was studied initially on the seventh day after onset. *(* ***A*** *)* ***PTA results of air conduction exam.*** The patient demonstrated a sensorineural hearing loss pattern in initial exam (left column) and had a hearing gain of less than the cut-off value for hearing improvement (10 dB; see method for definition) 1 month later (right column). Dashed and solid lines denote right and left ear threshold, respectively. *(* ***B*** *)* ***&*** *(* ***C*** *)* ***Neuromagnetic field patterns and source localizations.*** In initial MEG exam (left column), ECDs (green arrows) revealed a pattern of healthy-side dominance. In 1-month MEG exam (right column), the pattern became relatively symmetrical. Dipole sources (red dots) are localized at the auditory cortices of bilateral temporal lobes in patients'MRI images. MRI views are displayed according to neurological convention, i.e., subject' right hemisphere is on the right side of the images.

All participants underwent PTA exam to determine both air and bone conduction threshold, using test frequencies between 250 Hz to 8000 Hz. Controls had normal PTA results (threshold ≤ 25 dB HL for all frequencies). A unilateral sensorineural hearing loss was confirmed in all ISSNHL patients, characterized as cochlea being the lesion site based on results of reduced distortion-product otoacoustic emissions (DPOAEs) and within-normal-limit interaural latency differences for auditory brainstem responses (ABRs) [14]. Since for all patients, air and bone conduction thresholds were less than 65 dB HL at 1000 Hz (Figure 1, Table 1), the probing auditory stimulus was set at this frequency with an intensity of 75 dB SPL for the MEG exam. This moderate intensity was chosen to avoid further acoustic damage and cross-hearing contamination.

### MEG Paradigm

MEG measurements were done in a magnetically shielded room using a whole-head 306-channel neuromagnetometer (Vectorview™ 4-D Neuroimaging, Helsinki, Finland). Subjects sat upright with eyes open during measurements. Tone bursts (1000 Hz, 50 ms duration with 10 ms for ramp up and down, respectively, 75 dB SPL at the exit end of the plastic tube, with an interstimulus interval of four seconds) were delivered monaurally via molded earpieces using the SoundProbe™ program on a Macintosh computer. The contralateral (i.e. non-stimulated) ear was plugged by using molded earpiece to minimize the ear-to-ear crosstalk. Affected and healthy ears were monaurally stimulated in separate sessions separated by two minutes of rest. Trials with electro-oculographic amplitudes exceeding 150 *μ*V were rejected. MEG signals were sampled at 400 Hz and band-pass filtered at 0.03 to 100 Hz. About 90 artifact-free trials were averaged. Digital low-pass filtering at 30 Hz and high-pass filtering at 1 Hz was performed off-line. An equivalent current dipole (ECD) model consisting of bilateral sources was used to explain the MEG signals [15]. First, an initial guess of an independent source was done in both hemispheres respectively. Each ECD was applied to a subset of 40∼60 sensors around the maximum peak in one hemisphere with a goodness-of-fit (g) larger than 90% for acceptance. No magnetometers were included. Since the accuracy of dipole localization depends on the signal-to-noise ratio [16], we included a sensor only when the peak amplitude of the signal was stronger than 2 standard deviations above the baseline. After the ECD with the highest g value was identified, all channels were taken into account for further analysis so that it explained best the recorded magnetic field globally [15]. Peak latency was then extracted for these ECDs. T1-weighted MR images of subject brains were acquired using a 3.0 T Bruker MedSpec S300 system (Bruker, Kalsrube, Germany) for MEG-MRI co-registration. No obvious abnormality (e.g., vascular lesion, tumor growth, etc.) was found in those brain MRI-exams.

### Data Analysis

The epoch analyzed ranged from 50 ms before to 350 ms after stimulation onset. The prestimulus interval was used as baseline. The time window for N100m was 70∼160 ms [17], [18]. The inter-hemispheric differences of peak dipole amplitude and latency of N100m, observed in different hemispheres of controls and patients respectively, were evaluated using Wilcoxon signed rank test. Two kinds of ipsilateral/contralateral ratio (I/C) were used to assess the degree of hemispheric asymmetry: (1) I/C made by reactivity of different hemispheres to monaural stimulation (I/C_a_ and I/C_l_ for N100m dipole moment and latency, respectively), i.e., ratio of N100m in ipsilateral to that in contralateral hemisphere on unilateral-ear stimulation, and (2) I/C made by reactivity of ***same*** hemisphere to stimulus at two ears (I/C_as_ and I/C_ls_ for N100m dipole moment and latency, respectively), i.e., ratio of N100m in one hemisphere on ipsilateral-ear stimulation to that in the same hemisphere on contralateral-ear stimulation. The prognostic relevance of the hemispheric asymmetry as expressed in the relationship between I/C and hearing level/hearing gain in ISSNHL was evaluated using Spearman' rank correlation. Differences of I/C among prognostic subgroups (i.e., complete, partial, and no recovery) were evaluated using both Kruskal-Wallis and Mann-Whitney U test. Receiver operating characteristic (ROC) curve was then used to determine the best cut-off value of I/C for the prognostication of hearing improvement at the fixed stage. Statistical significance was thresholded at P<0.05.

## Results

In all subjects, N100m dipole was identifiable over each hemisphere and was localized bilaterally on the superior temporal planum with an orientation centrifugal to the auditory cortex (Figure 1, Table 2 and 3, Tables S1, S2, and S3). In the present study, both the healthy and affected ear was stimulated with the same stimulus. Although the same SPL sound corresponds to different HL sound in each patient with variable hearing loss, this did not jeopardize the interpretation of our results, since the analysis was based mainly on the ratio rather than the value of AEFs.

**Table 2:** Amplitude and latency of peak dipole moment for N100m.

	Control				ISSNHL patient							
					Initial				1 m			
	Left		Right		Healthy		Affected		Healthy		Affected	
Hemisphere	a	l	a	l	a	l	a	l	A	l	a	l
*Contralateral hemisphere*												
m	58.7	89.3	52.2	92.9	48.4	85.5	62.2	100.3	64.8	85.9	64.3	92.0
SD	27.7	10.9	13.1	11.9	18.6	14.1	29.5	24.2	28.6	8.2	35.6	14.6
*Ipsilateral hemisphere*												
m	32.8	103.4	42.9	102.6	57.9	92.0	31.1	109.8	65.8	92.2	46.2	101.7
SD	9.9	7.8	15.0	11.8	20.4	10.3	12.1	15.5	30.7	12.0	21.8	15.4

	a	l	a	l	a	l	a	l	A	l	a	l
*p_1_*	<0.001	<0.001(Left*/Right* pooled)			0.062	0.001(healthy*/affected* pooled)			0.015	<0.001		
*p_2_*					<0.001	0.677(healthy side dominance)			0.142	0.441		
*p_3_*	<0.001	<0.001	0.002	0.002	0.023	0.020	<0.001	0.024	0.520	0.002	0.005	0.002

Threshold for statistical significance using Wilcoxon signed rank test was set at P<0.05. Left, left-ear stimulation; Right, right-ear stimulation; Healthy, healthy-ear stimulation; Affected, affected-ear stimulation; Initial, initial MEG exam; 1 m, 1 month after initial exam (fixed stage); a, amplitude of N100m dipole moment (Q/nAm); l, latency of N100m dipole moment (ms); m, mean; sd, standard deviation; P1, significance of difference between pooled responses of contralateral vs. ipsilateral hemispheres on monaural stimulation to both ears in controls and patients, respectively; P2, significance of difference between pooled responses of hemispheres ipsilateral to vs. opposite to the healthy ears on monaural stimulation to both intact and affected ears of patients; P3, significance of difference between hemispheric responses on a subset level according to ear of stimulation (left-ear stimulation and right-ear stimulation respectively in controls, healthy-ear stimulation and affected-ear stimulation respectively in patients).

**Table 3:** Amplitude and latency of peak dipole moment for N100m (m±sd) in prognostic subgroups.

	ISSNHL patient							
	Initial				1 m			
	Healthy		Affected		Healthy		Affected	
Prognostic subgroups	a	l	a	L	a	l	a	l
*Contralateral hemisphere*								
c (n = 4)	47.8±14.1	88.5±19.6	88.1±21.1	85.8±9.6	52.7±12.1	83.9±9.7	70.7±41.1	83.8±13.2
p (n = 5)	45.5±23.5	81.7±9.5	41.2±31.3	123.1±28.3	60.5±41.8	84.8±6.3	58.7±57.8	93.6±19.5
n (n = 12)	49.9±19.2	86.0±14.7	62.4±25.7	95.6±20.3	69.3±25.8	85.7±8.6	66.4±18.1	92.7±12.3
*Ipsilateral hemisphere*								
c (n = 4)	64.1±23.5	92.6±12.2	34.1±6.5	95.9±1.7	76.0±40.4	89.4±6.5	41.0±17.6	94.8±5.8
p (n = 5)	59.4±16.2	90.5±10.9	25.5±15.9	123.7±8.7	61.5±38.7	97.9±18.5	44.9±32.3	106.1±25.5
n (n = 12)	55.3±22.1	92.5±10.3	32.5±12.0	108.7±15.8	64.1±26.0	90.8±10.3	48.4±19.6	102.2±12.6

Healthy, healthy-ear stimulation; Affected, affected-ear stimulation; Initial, initial MEG exam; 1 m, 1 month after initial exam (fixed stage); a, amplitude of N100m dipole moment (Q/nAm); l, latency of N100m dipole moment (ms); m, mean; sd, standard deviation; c, complete recovery; p, particl recovery; n, no recovery.

### Prognostic relevance of ipsilateral/contralateral ratio

**Figure 2:**
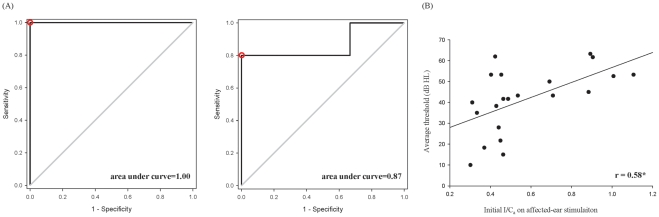
Prognostic effect of neuromagnetic index. (**A**) **ROC curves showed the best prediction effect of I/C for the hearing improvement.** ***Left, between subgroups of complete and partial recovery***, the optimal cut-off value was an initial I/C_ls_ on affected-ear stimulation at 1.34 (area under curve 1, sensitivity 100%, specificity 100%, red circle). ***Right, between subgroups of partial and no recovery***, the optimal cut-off value was an initial I/C_ls_ on healthy-ear stimulation at 0.76 (area under curve 0.87, sensitivity 80%, specificity 100%, red circle). (**B**) **Relationship between ipsilateral/contralateral ratio and hearing levels.** When ipsilateral/contralateral ratio were correlated to hearing levels, no significant correlation was revealed except for that between the initial I/C_a_ on affected-ear stimulation and the fixed hearing level (r = 0.58, p = 0.006). r, correlation coefficient; *, p<0.05.

About half of patients achieved hearing improvement (Table 1). Within-group differences of I/C among prognostic subgroups (i.e., complete, partial, and no recovery) evaluated using Kruskal-Wallis revealed statistical significance in three of them (Table 4 and 5): initial I/C_as_ on healthy-ear stimulation (p = 0.046), initial I/C_ls_ on healthy-ear stimulation (p = 0.027), and initial I/C_ls_ on affected-ear stimulation (p = 0.02). Mann-Whitney U test furthermore showed that the differences existed between subgroups of complete and partial recovery (p = 0.037 for I/C_as_ on healthy-ear stimulation, the smaller the ratio, the better recovery; p = 0.036 for I/C_ls_ on healthy-ear stimulation, the larger the ratio, the better recovery; p = 0.014 for I/C_ls_ on affected-ear stimulation, the smaller the ratio, the better recovery), as well as those of partial and no recovery (p = 0.027 for I/C_as_ on healthy-ear stimulation, the larger the ratio, the better recovery; p = 0.02 for I/C_ls_ on healthy-ear stimulation, the smaller the ratio, the better recovery; p = 0.026 for I/C_ls_ on affected-ear stimulation, the larger the ratio, the better recovery), but not those of complete and no recovery (p = 0.63 for I/C_as_ on healthy-ear stimulation; p = 0.28 for I/C_ls_ on healthy-ear stimulation; p = 0.25 for I/C_ls_ on affected-ear stimulation). The ROC curves in turn showed the best prediction effect of I/C for the hearing improvement at fixed stage: between subgroups of complete and partial recovery, the optimal cut-off value was an initial I/C_ls_ on affected-ear stimulation at 1.34 (area under curve 1, sensitivity 100%, specificity 100%); between subgroups of partial and no recovery, the optimal cut-off value was an initial I/C_ls_ on healthy-ear stimulation at 0.76 (area under curve 0.87, sensitivity 80%, specificity 100%; the smaller the ratio, the better recovery, Figure 2A).

**Table 4:** Ipsilateral/contralateral ratio of N100m.

									ISSNHL patient															
Control									Initial								1 m							
	Left				Right				Healthy				Affected				Healthy				Affected			
	I/C_a_	I/C_l_	I/C_as_	I/C_ls_	I/C_a_	I/C_l_	I/C_as_	I/C_ls_	I/C_a_	I/C_l_	I/C_as_	I/C_ls_	I/C_a_	I/C_l_	I/C_as_	I/C_ls_	I/C_a_	I/C_l_	I/C_as_	I/C_ls_	I/C_a_	I/C_l_	I/C_as_	I/C_ls_
1	0.46	1.21	0.92	1.14	1.60	1.04	0.80	1.10	1.08	1.11	0.91	1.04	0.46	1.13	0.55	1.21	0.84	1.09	1.24	1.07	1.49	1.09	1.01	1.11
2	0.86	0.97	0.50	0.89	0.86	1.04	1.47	1.13	3.17	1.00	1.75	0.69	0.31	1.08	0.56	1.58	2.87	1.06	0.96	1.07	0.40	0.91	1.19	0.90
3	0.42	1.24	0.48	1.14	0.70	0.98	0.62	1.07	2.64	0.94	0.83	1.11	0.30	0.94	0.96	0.80	1.72	1.00	0.84	0.95	0.43	1.00	0.88	1.05
4	0.33	1.27	0.75	1.16	0.94	0.97	0.41	1.06	0.57	1.27	2.26	0.76	0.90	0.91	0.23	1.53	0.80	1.17	1.11	0.98	1.13	1.00	0.82	1.19
5	0.39	1.19	0.41	1.07	0.68	1.09	0.65	1.21	1.36	0.88	1.07	1.03	0.89	1.00	1.13	0.86	0.76	1.09	0.95	0.98	0.98	1.10	0.78	1.22
6	0.77	1.14	0.94	1.12	0.83	1.08	0.68	1.09	0.68	1.17	1.07	0.86	0.43	1.22	0.27	1.67	0.80	1.05	1.74	0.81	0.85	1.25	0.39	1.62
7	0.22	1.38	0.35	1.31	0.74	1.22	0.46	1.28	2.42	1.05	1.04	0.82	0.42	1.02	0.98	1.30	2.01	1.04	1.08	0.99	0.28	1.00	0.52	1.05
8	1.08	1.31	0.64	1.15	0.35	1.00	0.59	1.14	1.10	1.22	0.58	0.91	0.33	1.01	0.63	1.36	0.85	1.05	0.49	0.94	0.91	0.94	1.56	1.04
9	1.07	0.91	0.79	1.07	0.57	1.17	0.78	0.98	1.18	1.19	1.83	0.75	0.71	1.04	0.46	1.66	0.76	1.65	1.82	1.42	2.82	1.28	1.18	1.50
10	0.42	1.10	0.48	0.82	0.70	1.05	0.62	1.41	1.01	1.41	1.52	0.75	1.01	0.86	0.67	1.60	0.47	1.05	1.06	1.12	0.89	1.39	0.39	1.30
11	1.49	1.05	0.85	1.29	0.92	1.20	1.62	0.97	1.15	1.16	0.76	0.99	0.69	0.99	1.05	1.15	0.66	1.00	0.56	0.88	0.85	1.00	1.01	1.13
12	0.86	1.45	0.72	1.02	1.25	0.82	1.49	1.17	1.06	1.10	0.79	0.94	0.45	1.11	0.61	1.30	0.84	1.18	1.41	1.06	0.74	0.98	0.44	1.09
13	0.53	1.10	0.66	1.14	0.70	1.24	0.56	1.19	1.18	1.15	1.62	1.15	0.49	1.44	0.36	1.44	1.03	1.20	0.49	1.09	0.34	1.11	0.71	1.22
14	0.59	1.09	0.56	1.32	0.82	1.25	0.87	1.03	1.18	1.00	0.85	1.10	0.40	1.22	0.56	1.11	1.19	1.00	1.74	0.85	0.86	1.10	0.59	1.29
15	0.50	1.16	0.71	1.09	1.10	1.09	0.78	1.16	1.04	1.09	0.86	1.08	0.37	1.18	0.45	1.19	3.13	1.10	1.88	1.10	0.52	1.20	0.86	1.20
16	0.47	1.19	0.65	1.09	1.09	1.09	0.78	1.19	1.01	1.09	0.86	1.07	0.54	1.35	0.63	1.38	0.88	1.08	0.80	1.01	0.67	0.97	0.74	1.04
17	0.54	1.42	0.31	1.29	0.55	1.47	0.98	1.62	1.81	1.02	0.72	1.20	0.46	1.52	1.16	1.30	0.64	1.00	0.95	1.19	0.75	1.29	0.51	1.08
18	0.96	1.08	0.83	1.08	0.90	1.08	1.04	1.08	1.49	0.91	4.01	0.57	1.11	0.87	0.41	1.39	1.15	0.98	3.14	0.74	1.26	1.13	0.46	1.51
19	0.94	1.07	0.57	1.07	0.74	1.16	1.22	1.16	1.23	0.92	0.66	1.13	0.44	1.33	0.83	1.08	1.09	0.92	0.77	1.10	0.82	1.30	1.17	1.08
20	0.62	1.18	0.84	1.18	0.97	1.11	0.72	1.11	1.01	1.10	0.38	1.07	0.45	1.26	1.19	1.30	0.65	1.09	0.65	1.20	0.39	1.30	0.39	1.18
21	0.38	1.10	0.43	1.17	0.65	1.27	0.43	1.17	0.53	1.11	0.48	1.02	0.88	1.19	0.99	1.29	0.92	1.04	0.86	0.98	0.42	1.27	0.45	1.35
m	0.66	1.17	0.64	1.12	0.84	1.11	0.84	1.16	1.33	1.09	1.18	0.95	0.57	1.13	0.70	1.31	1.14	1.09	1.17	1.02	0.85	1.12	0.76	1.20
SD	0.32	0.14	0.19	0.13	0.27	0.14	0.35	0.14	0.67	0.13	0.81	0.17	0.25	0.18	0.30	0.23	0.71	0.15	0.62	0.15	0.55	0.14	0.33	0.18
*p*									0.88	0.88	0.046†	0.027†	0.15	0.51	0.61	0.02†	0.79	0.72	0.45	0.30	0.55	0.90	0.90	0.82

	SSNHL patient															
	Initial								1 m							
	Healthy				Affected				Healthy				Affected			
Prognostic subgroups	I/C_a_	I/C_l_	I/C_as_	I/C_ls_	I/C_a_	I/C_l_	I/C_as_	I/C_ls_	I/C_a_	I/C_l_	I/C_as_	I/C_ls_	I/C_a_	I/C_l_	I/C_as_	I/C_ls_
*Mean*																
c (n = 4)	1.44	1.06	0.74	1.08	0.40	1.13	0.79	1.12	1.58	1.07	1.15	1.08	0.71	1.14	0.78	1.13
p (n = 5)	1.57	1.12	2.00	0.76	0.73	1.04	0.54	1.52	1.22	1.16	1.56	1.07	1.21	1.14	0.79	1.25
n (n = 12)	1.19	1.09	0.99	0.99	0.57	1.16	0.73	1.28	0.97	1.06	1.01	0.99	0.75	1.11	0.75	1.20
*Standard deviation*																
c (n = 4)	0.80	0.08	0.24	0.03	0.08	0.13	0.35	0.22	1.13	0.05	0.54	0.10	0.53	0.13	0.27	0.07
p (n = 5)	0.91	0.19	1.19	0.18	0.33	0.20	0.11	0.13	0.95	0.28	0.97	0.24	0.95	0.20	0.38	0.27
n (n = 12)	0.52	0.12	0.50	0.14	0.21	0.19	0.34	0.21	0.37	0.08	0.43	0.11	0.26	0.13	0.35	0.17

Threshold for statistical significance using Kruskal-Wallis test was set at P<0.05. Left, left-ear stimulation; Right, right-ear stimulation; Healthy, healthy-ear stimulation; Affected, affected-ear stimulation; Initial, initial MEG exam; 1 m, 1 month after initial exam (fixed stage); I/Ca, ipsilateral/contralateral ratio of N100m dipole moment amplitude in different hemispheres to monaural stimulation; I/Cl, ipsilateral/contralateral ratio of N100m dipole moment latency in different hemispheres to monaural stimulation; I/Cas, ipsilateral/contralateral ratio of N100m dipole moment amplitude in same hemisphere to stimulus at two ears; I/Cls, ipsilateral/contralateral ratio of N100m dipole moment latency in same hemisphere to stimulus at two ears; m, mean; sd, standard deviation; P, significance of difference in I/C among prognostic subgroups (i.e., complete, partial, and no recovery); †, significant difference.

**Table 5:**
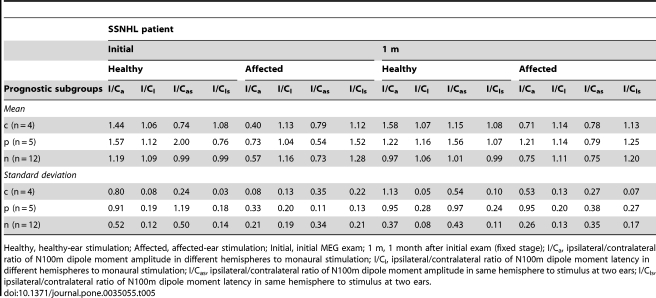
Ipsilateral/contralateral ratio of N100m in prognostic subgroups.

Healthy, healthy-ear stimulation; Affected, affected-ear stimulation; Initial, initial MEG exam; 1 m, 1 month after initial exam (fixed stage); I/C_a_, ipsilateral/contralateral ratio of N100m dipole moment amplitude in different hemispheres to monaural stimulation; I/C_l_, ipsilateral/contralateral ratio of N100m dipole moment latency in different hemispheres to monaural stimulation; I/C_as_, ipsilateral/contralateral ratio of N100m dipole moment amplitude in same hemisphere to stimulus at two ears; I/C_ls_, ipsilateral/contralateral ratio of N100m dipole moment latency in same hemisphere to stimulus at two ears.

When ipsilateral/contralateral ratio were correlated to hearing levels, no significant correlation was revealed except for that between the initial I/C_a_ on affected-ear stimulation and the hearing level at the fixed stage (r = 0.58, p = 0.006; the smaller the ratio, the lower the hearing level; Figure 2B) in ISSNHL. There was no correlation between hearing gain and ipsilateral/contralateral ratio at various stages.

### Interhemispheric differences of N100m

#### Normal-hearing subjects (Table 2 and S3)

When N100m activities of contralateral and ipsilateral hemispheres for all control subjects were respectively pooled from ear stimulation on both sides (42 measurements for each hemisphere), a contralateral dominance of dipole moment was noted (p<0.001). A faster N100m response was also noted in the contralateral hemisphere (p<0.001). A subset analysis (n = 21) of peak N100m moment made according to the ear stimulated revealed a significant contralateral preponderance upon both left-ear (p<0.001) and right-ear stimulation (p = 0.002). Inter-hemispheric latency differences were significant for both left-ear (p<0.001) and right-ear stimulation (p = 0.002) on the subset level.

#### ISSNHL patients (Table 2 and 3, Figure S1)

On initial MEG exam of patients, the contralateral N100m was significantly shorter in response latency (p = 0.001) as compared to that of ipsilateral hemisphere (n = 42). Contralateral dominance was not observed (p = 0.062) on the pooled data from stimulation of both ears (responses from the hemisphere opposite the stimulated healthy or deaf ear vs. those from the ipsilateral hemisphere; n = 42). However, a healthy-side dominance was observed when responses from hemispheres ipsilateral to the healthy ears were pooled and compared with those from hemispheres ipsilateral to the deaf ears, irrespective of the ear stimulated (p<0.001; n = 42). No inter-hemispheric difference in latency was observed (p = 0.677).

On MEG exam of patients at the fixed stage, the contralateral N100m was significantly shorter in response latency (p<0.001) as compared to that of ipsilateral hemisphere (n = 42). Contralateral dominance was observed (p = 0.015) on the pooled data from stimulation of both ears (n = 42). Neither evidence of “healthy-side dominance” (p = 0.142) nor inter-hemispheric difference in latency (p = 0.441) was observed when responses from hemispheres ipsilateral to the healthy ears were pooled and compared with those from hemispheres ipsilateral to the deaf ears, irrespective of the ear stimulated (n = 42).

## Discussion

One major and novel finding in the current study is the prognostic relevance of hemispheric asymmetry in terms of ipsilateral/contralateral ratio for N100m responses. When ipsilateral/contralateral ratio were grouped according to the prognosis and analyzed, the ROC curves demonstrated that the cut-off value with best prediction effect of hearing improvement at the fixed stage was an initial I/C_ls_ on affected-ear stimulation at 1.34 for the prognostication between subgroups of complete and partial recovery, and an initial I/C_ls_ on healthy-ear stimulation at 0.76 for that between subgroups of partial and no recovery, respectively (the smaller the ratio, the better recovery, Figure 2A). Strong correlation was furthermore noted only between the initial I/Ca on affected-ear stimulation and the hearing level at the fixed stage (positive correlation, i.e. the smaller the ratio, the lower the hearing level; Figure 2B) in ISSNHL. Since a lower hearing level at the fixed stage suggested a higher possibility for the patients to be recovered, a smaller initial I/C_a_ on affected-ear stimulation implied a bigger chance for hearing improvement in ISSNHL patients. It is noteworthy that the dipole moment of the N100m response in the contralateral hemisphere relative to affected-ear stimulation in initial MEG exam (62.2 nAm) was about the same to that of the N100m response in the contralateral hemisphere relative to healthy-ear stimulation in 1-mon MEG exam (64.8). This seems surprising given that the stimulus level used was constant. With a stimulus level of 75 dB SPL at 1000 Hz, the difference on hearing threshold translates into a pronounced disparity in sensation level (dB SL) of bilateral ears. Roughly, 65 dB HL translates to approximately 10 dB SL in the affected ear. For the healthy ear (no worse than 25 dB HL), the stimulus level is probably at least 50 dB SL. One reasonable explanation of this result should be the inner-ear hearing impairment itself. Previous study reported that inner-ear hearing loss revealed enhanced N100m response, and the amplitude of N100m response on affected-ear stimulation at 5∼10 dB SL was almost equal to that on healthy-ear stimulation at 50∼60 dB SL [8]. The finding thus justified the conjecture that cochlea might be the most probable lesion site of ISSNHL.

The above-mentioned findings thus pinpoint a good prognostication of the outcome at defined time. Since the ipsilateral/contralateral ratio subserves an indicator of auditory plasticity [9], a smaller initial I/C_ls_ on either affected- or healthy-ear stimulation and I/C_a_ on affected-ear stimulation in ISSNHL might entail a better adaptation and compensational bi-hemispheric synergism of the auditory system to the abrupt hearing loss for the functional restitution after the insult. The observations in the current study suggested that the ipsilateral/contralateral ratio of both N100m latency and amplitude be a sensitive parameter to index for the subtle functional modulation/plasticity in ISSNHL of slight to moderate degree. Although efforts have been made to correlate various variables with the prognosis, no single biomarker was yet found to reliably prognosticate the eventual outcome and/or hearing level in ISSNHL [4], [13], [19], [20], [21], [22]. The aforementioned heterogeneity between neuromagnetic index and prognosis in the present study (i.e. the best parameter was different between subgroups of complete and partial recovery, and between subgroups of partial and no recovery) possibly echoed different pathogeneses of ISSNHL.

The actual mechanisms of central auditory plasticity in the functional recovery of ISSNHL are currently unknown. Although the mean age of our controls is less than that of patients, the observations cannot be ascribed to the ageing effect or to the influence of dipole location [5], [6], [23], [24]. One plausible explanation for the prognostication effect of initial I/C on either affected- or healthy-ear stimulation in patients, is that the effect of cochlear lesion(s) might be bilateral through retrocochlear crossing fibers, and thus causing a changes of inter-hemispheric inhibition [5], [6], [24]. The effect, probably coupled with changes in neurotransmission/neuromodulation, might also implicate the extent of peripheral injuries and launch a corresponding reorganization for the mending in central auditory pathway [2], [25]. Such reorganization would help avoid further deterioration in the auditory system resulting from cessation of electrical and nutritional input due to cochlear damages [2], [25].

In the present study, elapsed time for the initial MEG exam after disease onset ranged from 3 days to 3 weeks. It seemed that this could lead to an underestimate of the difference between findings at initial and fixed stages in patients who had late initial scan at 3 weeks, though the test interval between two stages was actually the same among patients. However, MEG results were significantly different between the two stages. The significantly higher initial ipsilateral/contralateral ratio on healthy-ear stimulation and the significantly lower initial ipsilateral/contralateral ratio on affected-ear stimulation, in comparison to that of controls on either-ear stimulation, yielded a pattern of healthy-side dominance in the acute stage of ISSNHL. Higher ipsilateral/contralateral ratio on healthy-ear stimulation and lower ipsilateral/contralateral ratio on affected-ear stimulation at initial MEG exam for patients were both replaced by values approaching ∼1.0 at the fixed stage, resulting in a relatively symmetrical pattern of N100m responses. This finding confirmed previous results showing a pattern of more equal activation over bilateral hemispheres on monaural stimulation in chronically unilateral deaf patients than those in acute stage and normal-hearing subjects [9], [26], [27], [28]. Our finding is also in cooperative with the aforementioned fMRI study [7], in which the pattern of “ipsilateral dominance” on healthy-ear stimulation in the acute stage of ISSNHL showed a tendency to become balanced after partial recovery of hearing function one month later.

One possible explanation of our finding is the involvement of neurochemical changes observed in the auditory system following peripheral hearing loss. Animal studies have revealed a down-regulation of both ipsilateral excitatory receptor expression/binding and contralateral inhibitory neurotransmitters synthesis with respect to the affected ear in the central auditory pathway [29], [30]. Levels of these altered receptors/neurotransmitters came near a relatively equal range between bilateral pathways after the cochlear damage, with or without repair of the peripheral damage [29], [30]. Such changes thus underpin the reversal from healthy-side dominance to a more balanced activation of both auditory cortices as we observed in the fixed stage of ISSNHL.

In summary, the hemispheric asymmetry expression at initial stages bears the best prognostic potential for the treatment outcomes and/or the hearing levels at the fixed stage in acute unilateral ISSNHL of mild-to-moderate degree. In addition, this study suggested that a dynamic process of central auditory plasticity can be induced by peripheral lesions. Our study demonstrated that such brain signature of central auditory plasticity at defined time can serve as a prognostication predictor for ISSNHL. To strengthen the clinical application of our findings, studies using electroencephalography are required to verify the temporal changes in asymmetry expression within clinical settings since MEG is mainly a research tool.

## Supporting Information

Figure S1 **Source waveforms at respective stages by ear stimulation in ISSNHL patients.** Healthy-side dominance of N100m responses was observed initially (red line, initial MEG exam). At the fixed stage (green line, 1 month after initial exam), a relatively symmetrical pattern (or even contralateral dominance) of N100m responses was noted. Healthy, healthy-ear stimulation; Affected, affected-ear stimulation; Contra, hemisphere contralateral to the stimulated ear; Ipsi, hemisphere ipsilateral to the stimulated ear.
(TIF)

Table S1 **Amplitude and latency of N100m dipole moment on initial MEG exam and 1 month after initial exam in controls.** The excellent consistence of MEG makes it suitable for repeated follow-up measurements of auditory evoked responses, which is in line with experiences in our lab. To verify that the findings observed in our patients did not result from the differences due simply to the test-retest bias, we surveyed again the test-retest reliability for six additional controls with the same test-interval (i.e. one month) applied in ISSNHL patients. The results of evaluation for the reliability of repeated MEG exams in these normal hearing subjects showed no significant differences between test and retest over an interval of about one month in terms of peak dipole moment amplitude and peak latency.
(DOC)

Table S2 **Relative position of N100m peak dipole at various stages in terms of Talairach coordinates** (**x, y, z, in mm**). Relative position of N100m peak dipole between 2 repeated measurements in ISSNHL was expressed in terms of Talairach' nomenclature. Differences of N100m dipole location (x, y, and z coordinates, respectively) at various stages were evaluated using Wilcoxon signed rank test. There are no significant differences between N100m source locations of two stages.
(DOC)

Table S3 **Amplitude and latency of peak dipole moment for N100m.**
(DOC)

## References

[1] Dijkhuizen RM, Singhal AB, Mandeville JB, Wu O, Halpern EF (2003). Correlation between brain reorganization, ischemic damage, and neurologic status after transient focal cerebral ischemia in rats: a functional magnetic resonance imaging study.. J Neurosci.

[2] Durham D, Park DL, Girod DA (2000). Central nervous system plasticity during hair cell loss and regeneration.. Hear Res.

[3] Byl FM (1984). Sudden hearing loss: eight years' experience and suggested prognostic table.. Laryngoscope.

[4] Harada T (1996). Patterns of hearing recovery in idiopathic sudden sensorineural hearing loss.. Br J Audiol.

[5] Po-Hung Li L, Shiao AS, Lin YY, Chen LF, Niddam DM (2003). Healthy-side dominance of cortical neuromagnetic responses in sudden hearing loss.. Ann Neurol.

[6] Li LPH, Shiao AS, Chen LF, Niddam DM, Chang SY (2006). Healthy-side dominance of middle- and long-latency neuromagnetic fields in idiopathic sudden sensorineural hearing loss.. Eur J Neurosci.

[7] Suzuki M, Kouzaki H, Nishida Y, Shiino A, Ito R (2002). Cortical representation of hearing restoration in patients with sudden deafness.. Neuroreport.

[8] Morita T, Hiraumi H, Fujiki N, Naito Y, Nagamine T (2007). A recovery from enhancement of activation in auditory cortex of patients with idiopathic sudden sensorineural hearing loss.. Neurosci Res.

[9] Ponton CW, Vasama JP, Tremblay K, Khosla D, Kwong B (2001). Plasticity in the adult human central auditory system: evidence from late-onset profound unilateral deafness.. Hear Res.

[10] Wilson WR, Byl FM, Laird N (1980). The efficacy of steroids in the treatment of idiopathic sudden hearing loss. A double-blind clinical study.. Arch Otolaryngol.

[11] Murai K, Tsuiki T, Kusano H, Shishido K (1994). Clinical study of audiograms in the initial stage and fixed stage of sudden deafness.. Acta.

[12] Ochi K, Ohashi T, Kenmochi M (2003). Hearing impairment and tinnitus pitch in patients with unilateral tinnitus: comparison of sudden hearing loss and chronic tinnitus.. Laryngoscope.

[13] Mamak A, Yilmaz S, Cansiz H, Inci E, Guclu E (2005). A study of prognostic factors in sudden hearing loss.. Ear Nose Throat J.

[14] Gstoettner W, Neuwirth-Riedl K, Swoboda H, Mostbeck W, Burian M (1992). Specificity of auditory brainstem response audiometry criteria in acoustic neuroma screening as a function of deviations of reference values in patients with cochlear hearing loss.. Eur Arch Otorhinolaryngol.

[15] Hari R, Makela JP (1988). Modification of neuromagnetic responses of the human auditory cortex by masking sounds.. Exp Brain Res.

[16] Jacobson GP (1994). Magnetoencephalographic studies of auditory system function.. J Clin Neurophysiol.

[17] Woldorff MG, Tempelmann C, Fell J, Tegeler C, Gaschler-Markefski B (1999). Lateralized auditory spatial perception and the contralaterality of cortical processing as studied with functional magnetic resonance imaging and magnetoencephalography.. Hum Brain Mapp.

[18] Kanno A, Nakasato N, Murayama N, Yoshimoto T (2000). Middle and long latency peak sources in auditory evoked magnetic fields for tone bursts in humans.. Neurosci Lett.

[19] Fetterman BL, Saunders JE, Luxford WM (1996). Prognosis and treatment of sudden sensorineural hearing loss.. Am J Otol.

[20] Hirano K, Ikeda K, Kawase T, Oshima T, Kekehata S (1999). Prognosis of sudden deafness with special reference to risk factors of microvascular pathology.. Auris Nasus Larynx.

[21] Hoth S (2005). On a possible prognostic value of otoacoustic emissions: a study on patients with sudden hearing loss.. Eur Arch Otorhinolaryngol.

[22] Narozny W, Kuczkowski J, Kot J, Stankiewicz C, Sicko Z (2006). Prognostic factors in sudden sensorineural hearing loss: our experience and a review of the literature.. Ann Otol Rhinol Laryngol.

[23] Pekkonen E, Huotilainen M, Virtanen J, Sinkkonen J, Rinne T (1995). Age-related functional differences between auditory cortices: a whole-head MEG study.. Neuroreport.

[24] Hsieh JC, Cheng H, Hsieh HM, Liao KK, Wu YT (2002). Loss of interhemispheric inhibition on the ipsilateral primary sensorimotor cortex in patients with brachial plexus injury: fMRI study.. Ann Neurol.

[25] Illing RB, Reisch A (2006). Specific plasticity responses to unilaterally decreased or increased hearing intensity in the adult cochlear nucleus and beyond.. Hear Res.

[26] Scheffler K, Bilecen D, Schmid N, Tschopp K, Seelig J (1998). Auditory cortical responses in hearing subjects and unilateral deaf patients as detected by functional magnetic resonance imaging.. Cereb Cortex.

[27] Vasama JP, Makela JP, Parkkonen L, Hari R (1994). Auditory cortical responses in humans with congenital unilateral conductive hearing loss.. Hear Res.

[28] Fujiki N, Naito Y, Nagamine T, Shiomi Y, Hirano S (1998). Influence of unilateral deafness on auditory evoked magnetic field.. Neuroreport.

[29] Mossop JE, Wilson MJ, Caspary DM, Moore DR (2000). Down-regulation of inhibition following unilateral deafening.. Hear Res.

[30] Sato K, Shiraishi S, Nakagawa H, Kuriyama H, Altschuler RA (2000). Diversity and plasticity in amino acid receptor subunits in the rat auditory brain stem.. Hear Res.

