# Supplementary figures and images for: Neuromagnetic Index of Hemispheric Asymmetry Prognosticating the Outcome of Sudden Hearing Loss
Source: PLoS One. 2012 Apr 20;7(4):e35055. doi: 10.1371/journal.pone.0035055 (PMC3332152; doi:10.1371/journal.pone.0035055)

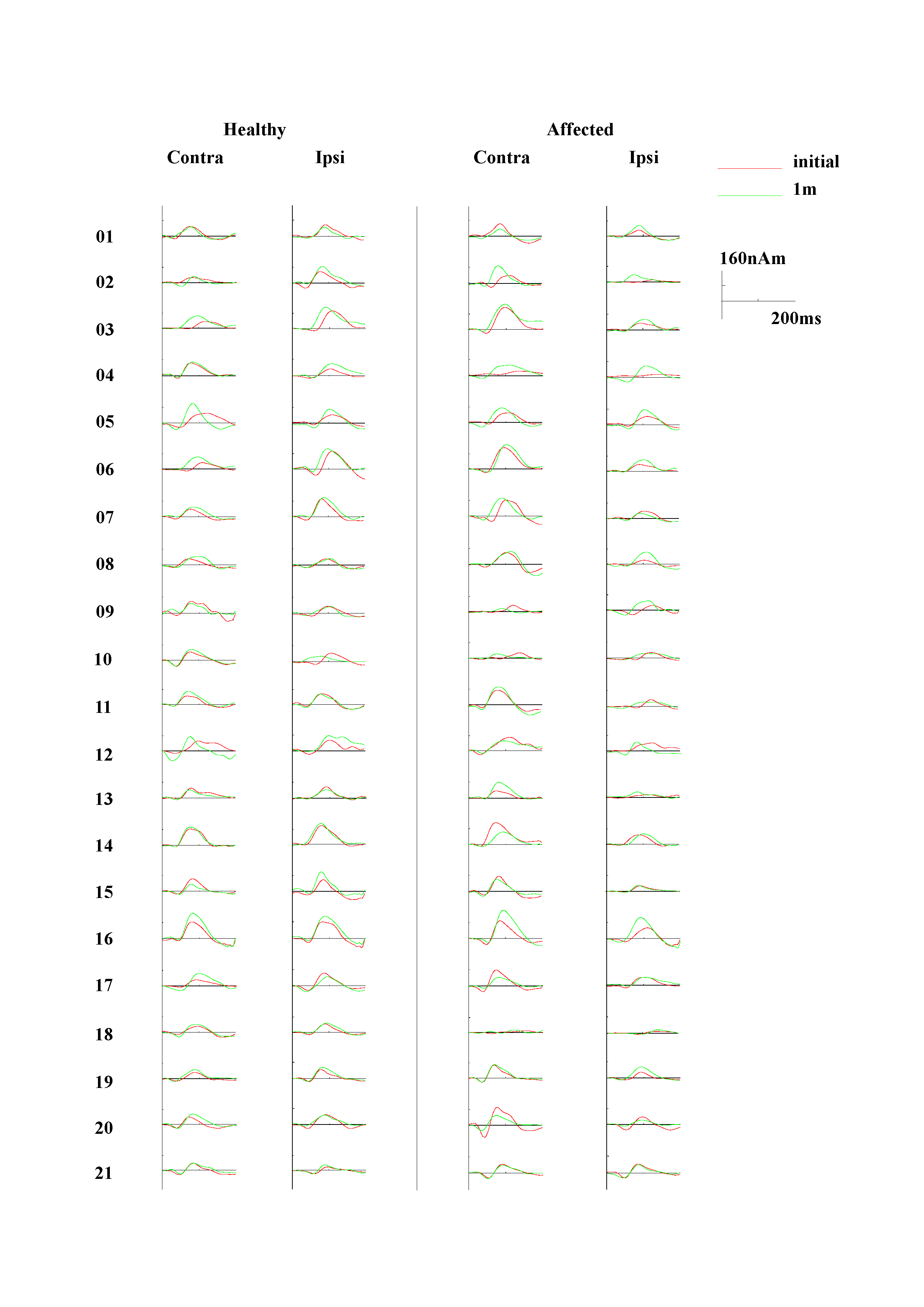

Supplement: Figure S1 — Source waveforms at respective stages by ear stimulation in ISSNHL patients. Healthy-side dominance of N100m responses was observed initially (red line, initial MEG exam). At the fixed stage (green line, 1 month after initial exam), a relatively symmetrical pattern (or even contralateral dominance) of N100m responses was noted. Healthy, healthy-ear stimulation; Affected, affected-ear stimulation; Contra, hemisphere contralateral to the stimulated ear; Ipsi, hemisphere ipsilateral to the stimulated ear. (TIF) [file pone.0035055.s001.tif]
