# Supplementary material for: Neuromagnetic Index of Hemispheric Asymmetry Prognosticating the Outcome of Sudden Hearing Loss
Source: PLoS One. 2012 Apr 20;7(4):e35055. doi: 10.1371/journal.pone.0035055 (PMC3332152; doi:10.1371/journal.pone.0035055)
Supplement: Table S1 — Amplitude and latency of N100m dipole moment on initial MEG exam and 1 month after initial exam in controls. The excellent consistence of MEG makes it suitable for repeated follow-up measurements of auditory evoked responses, which is in line with experiences in our lab. To verify that the findings observed in our patients did not result from the differences due simply to the test-retest bias, we surveyed again the test-retest reliability for six additional controls with the same test-interval (i.e. one month) applied in ISSNHL patients. The results of evaluation for the reliability of repeated MEG exams in these normal hearing subjects showed no significant differences between test and retest over an interval of about one month in terms of peak dipole moment amplitude and peak latency. (DOC) [file pone.0035055.s002.doc]

**Table S1.** **Amplitude and latency of N100m dipole moment on initial MEG exam and 1 month after initial exam in controls.**

| **Table S1. Amplitude and latency of N100m dipole moment on initial MEG exam and 1 month after initial exam in controls.** | | | | | | | | | | | | | |  |
| --- | --- | --- | --- | --- | --- | --- | --- | --- | --- | --- | --- | --- | --- | --- |
|  |  |  |  |  |  |  |  | Control |  |  |  |  |  |  |
| Hemisphere |  |  | Contralateral | | | | |  | Ipsilateral | | | | |  |
|  |  |  | Left | |  | Right | |  | Left | |  | Right | |  |
|  | Gender | Age | a | l |  | a | l |  | a | l |  | a | l |  |
| *Initial* |  |  |  |  |  |  |  |  |  |  |  |  |  |  |
| 1 | M | 29 | 64.7 | 81.3 |  | 99.2 | 86.0 |  | 41.6 | 86.0 |  | 83.4 | 99.2 |  |
| 2 | F | 21 | 56.4 | 90.1 |  | 46.0 | 92.1 |  | 48.1 | 97.1 |  | 38.1 | 101.0 |  |
| 3 | F | 27 | 72.0 | 97.0 |  | 51.2 | 78.3 |  | 51.0 | 87.9 |  | 66.7 | 97.0 |  |
| 4 | M | 25 | 64.0 | 85.2 |  | 99.0 | 86.0 |  | 42.3 | 86.0 |  | 83.4 | 99.4 |  |
| 5 | M | 24 | 34.7 | 86.0 |  | 32.9 | 90.5 |  | 44.4 | 100.2 |  | 27.0 | 94.0 |  |
| 6 | F | 25 | 71.0 | 104.1 |  | 69.3 | 99.0 |  | 59.3 | 101.0 |  | 68.9 | 116.1 |  |
| m |  |  | 60.5 | 90.6 |  | 66.3 | 88.7 |  | 47.8 | 93.0 |  | 61.3 | 101.1 |  |
| SD |  |  | 27.6 | 17.0 |  | 56.0 | 14.0 |  | 13.4 | 14.3 |  | 47.1 | 15.5 |  |
| *1 m* |  |  |  |  |  |  |  |  |  |  |  |  |  |  |
| 1 |  |  | 67.2 | 85.6 |  | 95.2 | 82.0 |  | 42.8 | 86.0 |  | 78.4 | 94.1 |  |
| 2 |  |  | 58.9 | 90.0 |  | 40.0 | 97.0 |  | 46.0 | 90.7 |  | 39.3 | 105.0 |  |
| 3 |  |  | 73.5 | 93.2 |  | 53.3 | 81.1 |  | 52.0 | 89.9 |  | 63.0 | 99.8 |  |
| 4 |  |  | 68.0 | 89.1 |  | 95.0 | 82.0 |  | 43.0 | 86.8 |  | 78.4 | 94.0 |  |
| 5 |  |  | 34.4 | 88.0 |  | 37.7 | 94.2 |  | 41.8 | 101.0 |  | 23.3 | 94.9 |  |
| 6 |  |  | 71.0 | 101.0 |  | 67.4 | 102.0 |  | 55.8 | 101.9 |  | 72.5 | 118.0 |  |
| m |  |  | 62.2 | 91.2 |  | 64.8 | 89.7 |  | 46.9 | 92.7 |  | 59.2 | 101.0 |  |
| SD |  |  | 14.5 | 5.4 |  | 25.8 | 9.1 |  | 5.7 | 7.0 |  | 22.9 | 9.4 |  |
| *p* |  |  | 0.078 | 0.600 |  | 0.345 | 0.498 |  | 0.462 | 0.752 |  | 0.114 | 0.917 |  |
| Threshold for statistical significance using Wilcoxon signed rank test was set at P < 0.05. Left, left-ear stimulation; Right, right-ear stimulation; Initial, initial MEG exam; 1 m, 1 month after initial exam; a, amplitude of N100m dipole moment (Q/nAm); l, latency of N100m dipole moment (ms); m, mean; sd, standard deviation; P, significance of difference between responses of initial MEG exam vs. 1 month after initial exam on monaural stimulation to both ears. | | | | | | | | | | | | | | |
