# Supplementary material for: Neuromagnetic Index of Hemispheric Asymmetry Prognosticating the Outcome of Sudden Hearing Loss
Source: PLoS One. 2012 Apr 20;7(4):e35055. doi: 10.1371/journal.pone.0035055 (PMC3332152; doi:10.1371/journal.pone.0035055)
Supplement: Table S2 — Relative position of N100m peak dipole at various stages in terms of Talairach coordinates (x, y, z, in mm). Relative position of N100m peak dipole between 2 repeated measurements in ISSNHL was expressed in terms of Talairach' nomenclature. Differences of N100m dipole location (x, y, and z coordinates, respectively) at various stages were evaluated using Wilcoxon signed rank test. There are no significant differences between N100m source locations of two stages. (DOC) [file pone.0035055.s003.doc]

**Table S2. Relative position of N100m peak dipole at various stages in terms of Talairach coordinates (x, y, z, in mm).**

| **Table S2. Relative position of N100m peak dipole at various stages in terms of Talairach coordinates (*x, y, z,* in mm).** | | | | | | | | | | | | | | | | | | | | | | | |
| --- | --- | --- | --- | --- | --- | --- | --- | --- | --- | --- | --- | --- | --- | --- | --- | --- | --- | --- | --- | --- | --- | --- | --- |
|  | Talairach coordinates | | | | | | | | | | | | | | | | | | | | | | |
|  | Left | | | | | | | | | | |  | Right | | | | | | | | | | |
|  | *x* | | |  | *y* | | |  | *z* | | |  | *x* | | |  | *y* | | |  | *z* | | |
| Stages | L |  | R |  | L |  | R |  | L |  | R |  | L |  | R |  | L |  | R |  | L |  | R |
| *Initial* | |  |  |  |  |  |  |  |  |  |  |  |  |  |  |  |  |  |  |  |  |  |  |
| 1 | -54.2 |  | 44.8 |  | -21.6 |  | -26.0 |  | 6.5 |  | 9.8 |  | -54.0 |  | 49.3 |  | -22.4 |  | -22.7 |  | 6.4 |  | 8.0 |
| 2 | -39.9 |  | 53.9 |  | -25.2 |  | -25.6 |  | 12.1 |  | 14.7 |  | -36.5 |  | 45.6 |  | -29.8 |  | -24.9 |  | 18.2 |  | 7.6 |
| 3 | -56.6 |  | 55.1 |  | -17.9 |  | -18.4 |  | 5.9 |  | 7.1 |  | -55.7 |  | 53.0 |  | -21.6 |  | -30.7 |  | 14.5 |  | 10.1 |
| 4 | -36.2 |  | 44.8 |  | -30.2 |  | -26.4 |  | 18.2 |  | 6.8 |  | -38.2 |  | 41.3 |  | -23.6 |  | -29.9 |  | 14.5 |  | 9.5 |
| 5 | -52.1 |  | 49.5 |  | -28.7 |  | -22.9 |  | -13.1 |  | 7.4 |  | -56.4 |  | 47.8 |  | -19.5 |  | -18.6 |  | 7.0 |  | 13.1 |
| 6 | -51.7 |  | 51.7 |  | -18.2 |  | -16.0 |  | 14.0 |  | 11.7 |  | -52.6 |  | 49.6 |  | -23.4 |  | -24.8 |  | 11.9 |  | 14.1 |
| 7 | -40.3 |  | 52.1 |  | -22.8 |  | -21.0 |  | 14.7 |  | 10.5 |  | -42.4 |  | 59.9 |  | -25.2 |  | -19.2 |  | 17.3 |  | 4.1 |
| 8 | -53.9 |  | 53.0 |  | -31.5 |  | -31.0 |  | 17.7 |  | 14.8 |  | -55.4 |  | 55.6 |  | -26.7 |  | -28.4 |  | 18.3 |  | 17.8 |
| 9 | -49.6 |  | 41.7 |  | -25.7 |  | -23.8 |  | 9.6 |  | 14.3 |  | -47.3 |  | 44.0 |  | -26.5 |  | -29.3 |  | 9.3 |  | 18.1 |
| 10 | -45.0 |  | 55.1 |  | -29.1 |  | -18.4 |  | 6.5 |  | 7.7 |  | -39.6 |  | 55.4 |  | -30.5 |  | -18.0 |  | 11.8 |  | 6.3 |
| 11 | -64.1 |  | 41.2 |  | -16.8 |  | -29.9 |  | 14.7 |  | 17.8 |  | -56.9 |  | 40.6 |  | -18.3 |  | -29.7 |  | 6.1 |  | 16.4 |
| 12 | -45.9 |  | 52.9 |  | -25.0 |  | -31.2 |  | 8.0 |  | 18.7 |  | -50.2 |  | 49.9 |  | -14.5 |  | -27.6 |  | 11.7 |  | 15.1 |
| 13 | -51.7 |  | 47.2 |  | -17.2 |  | -18.7 |  | 12.8 |  | 11.7 |  | -49.2 |  | 49.2 |  | -17.1 |  | -28.7 |  | 11.3 |  | 21.6 |
| 14 | -49.6 |  | 41.7 |  | -25.7 |  | -23.8 |  | 6.6 |  | 14.3 |  | -47.3 |  | 44.0 |  | -26.5 |  | -29.3 |  | 9.3 |  | 18.1 |
| 15 | -58.9 |  | 43.2 |  | -31.2 |  | -29.9 |  | 7.6 |  | 16.2 |  | -57.7 |  | 39.0 |  | -29.7 |  | -29.7 |  | 8.0 |  | 16.8 |
| 16 | -41.6 |  | 43.1 |  | -30.1 |  | -21.7 |  | 15.1 |  | 12.6 |  | -48.9 |  | 55.2 |  | -28.0 |  | -28.0 |  | 15.8 |  | 18.1 |
| 17 | -56.5 |  | 44.8 |  | -18.7 |  | -25.8 |  | 7.3 |  | 10.9 |  | -45.9 |  | 53.9 |  | -25.9 |  | -19.3 |  | 11.3 |  | 6.4 |
| 18 | -55.0 |  | 41.7 |  | -27.0 |  | -33.9 |  | 12.0 |  | 10.5 |  | -50.8 |  | 51.9 |  | -24.8 |  | -24.6 |  | 7.9 |  | 9.5 |
| 19 | -44.0 |  | 50.4 |  | -28.3 |  | -25.6 |  | 7.4 |  | 9.5 |  | -51.9 |  | 39.1 |  | -30.4 |  | -31.0 |  | 18.0 |  | 6.6 |
| 20 | -50.0 |  | 55.1 |  | -26.1 |  | -14.8 |  | 6.5 |  | 11.7 |  | -39.6 |  | 55.4 |  | -29.5 |  | -18.0 |  | 11.8 |  | 10.3 |
| 21 | -58.9 |  | 43.2 |  | -31.2 |  | -22.9 |  | 7.6 |  | 16.2 |  | -57.7 |  | 41.1 |  | -29.7 |  | -30.0 |  | 8.0 |  | 6.6 |
| m | -50.3 |  | 47.9 |  | -25.2 |  | -24.2 |  | 9.4 |  | 12.1 |  | -49.2 |  | 48.6 |  | -24.9 |  | -25.8 |  | 11.8 |  | 12.1 |
| SD | 7.2 |  | 5.2 |  | 5.0 |  | 5.2 |  | 6.5 |  | 3.5 |  | 6.8 |  | 6.3 |  | 4.7 |  | 4.7 |  | 4.0 |  | 5.2 |
| *1 mon* |  |  |  |  |  |  |  |  |  |  |  |  |  |  |  |  |  |  |  |  |  |  |  |
| 1 | -42.5 |  | 45.4 |  | -30.1 |  | -26.3 |  | 7.3 |  | 9.6 |  | -58.8 |  | 49.1 |  | -29.9 |  | -24.3 |  | 7.3 |  | 6.1 |
| 2 | -49.0 |  | 51.5 |  | -23.1 |  | -22.9 |  | 10.1 |  | 13.7 |  | -37.2 |  | 46.5 |  | -34.2 |  | -24.7 |  | -18.2 |  | 9.8 |
| 3 | -61.2 |  | 54.3 |  | -27.3 |  | -18.6 |  | 15.2 |  | 6.1 |  | -55.7 |  | 54.3 |  | -28.4 |  | -18.1 |  | 16.0 |  | 12.3 |
| 4 | -41.4 |  | 45.8 |  | -30.9 |  | -24.9 |  | 15.7 |  | 17.9 |  | -39.2 |  | 44.3 |  | -30.5 |  | -22.7 |  | 17.5 |  | 13.3 |
| 5 | -44.4 |  | 48.5 |  | -26.6 |  | -17.9 |  | 6.9 |  | -11.2 |  | -52.1 |  | 51.1 |  | -19.6 |  | -19.1 |  | 8.2 |  | 12.1 |
| 6 | -56.9 |  | 51.0 |  | -28.5 |  | -30.4 |  | 17.9 |  | 16.2 |  | -58.6 |  | 54.2 |  | -26.0 |  | -31.2 |  | 17.8 |  | 17.2 |
| 7 | -55.6 |  | 48.2 |  | -16.2 |  | -27.3 |  | 9.2 |  | 16.3 |  | 57.3 |  | -47.4 |  | -15.9 |  | -27.9 |  | 7.3 |  | 13.4 |
| 8 | -60.7 |  | 57.9 |  | -25.1 |  | 26.9 |  | 17.6 |  | 16.9 |  | -51.5 |  | 49.7 |  | -29.7 |  | -28.0 |  | 11.9 |  | 16.9 |
| 9 | -53.9 |  | 40.6 |  | -21.3 |  | -23.6 |  | 6.9 |  | 11.3 |  | -44.9 |  | 43.8 |  | -30.1 |  | -23.3 |  | 10.8 |  | 11.9 |
| 10 | -51.3 |  | 52.2 |  | -24.8 |  | -20.6 |  | 9.0 |  | 7.3 |  | -48.9 |  | 53.8 |  | -24.6 |  | -19.6 |  | 9.8 |  | 11.0 |
| 11 | -46.9 |  | 47.4 |  | -27.4 |  | -29.1 |  | 10.4 |  | 8.9 |  | -48.5 |  | 53.5 |  | -27.4 |  | -21.2 |  | 9.8 |  | 7.4 |
| 12 | -58.3 |  | 47.8 |  | -23.5 |  | -26.1 |  | 13.8 |  | 18.7 |  | -58.0 |  | 45.3 |  | -16.1 |  | -33.9 |  | 14.7 |  | 17.3 |
| 13 | -55.8 |  | 53.0 |  | -20.2 |  | -14.5 |  | 13.7 |  | 12.4 |  | -57.0 |  | 54.1 |  | -18.1 |  | -31.4 |  | 9.7 |  | 18.8 |
| 14 | -53.9 |  | 40.6 |  | -22.3 |  | -23.6 |  | 7.9 |  | 11.3 |  | -44.9 |  | 43.8 |  | -30.1 |  | -23.3 |  | 10.8 |  | 11.9 |
| 15 | -52.8 |  | 44.9 |  | -20.9 |  | -20.2 |  | 9.2 |  | 14.1 |  | -51.8 |  | 42.9 |  | -23.2 |  | -30.9 |  | 8.1 |  | 17.7 |
| 16 | -39.0 |  | 44.6 |  | -23.3 |  | -26.0 |  | 12.4 |  | 7.3 |  | -39.2 |  | 51.2 |  | -31.7 |  | -21.9 |  | 6.2 |  | 7.9 |
| 17 | -53.0 |  | 56.4 |  | -16.5 |  | -18.2 |  | 14.0 |  | 12.1 |  | -40.7 |  | 48.0 |  | -23.9 |  | -23.7 |  | 11.8 |  | 7.6 |
| 18 | -56.7 |  | 41.3 |  | -18.4 |  | -30.3 |  | 6.2 |  | 9.1 |  | -53.6 |  | 48.9 |  | -22.1 |  | -16.6 |  | 9.4 |  | 13.5 |
| 19 | -47.3 |  | 45.4 |  | -25.7 |  | -34.2 |  | 18.3 |  | 11.5 |  | -47.4 |  | 36.5 |  | -27.3 |  | -33.3 |  | -7.4 |  | 15.8 |
| 20 | -51.3 |  | 52.2 |  | -24.8 |  | -20.6 |  | 9.0 |  | 7.3 |  | -48.9 |  | 53.8 |  | -25.6 |  | -19.6 |  | 9.8 |  | 7.0 |
| 21 | -52.8 |  | 44.9 |  | -21.9 |  | -27.2 |  | 11.2 |  | 10.1 |  | -51.8 |  | 50.9 |  | -20.2 |  | -15.6 |  | 8.1 |  | 15.1 |
| m | -51.7 |  | 48.3 |  | -23.8 |  | -21.7 |  | 11.5 |  | 10.8 |  | -44.3 |  | 44.2 |  | -25.5 |  | -24.3 |  | 8.5 |  | 12.6 |
| SD | 6.2 |  | 5.0 |  | 4.0 |  | 12.2 |  | 3.9 |  | 6.3 |  | 24.2 |  | 21.5 |  | 5.3 |  | 5.5 |  | 8.0 |  | 3.9 |
| *p* | 0.34 |  | 0.93 |  | 0.24 |  | 0.74 |  | 0.24 |  | 0.26 |  | 0.68 |  | 0.9 |  | 0.59 |  | 0.59 |  | 0.66 |  | 0.57 |
| Threshold for statistical significance using Wilcoxon signed rank test was set at P < 0.05. Left, left-ear stimulation; Right, right-ear stimulation; L, left hemisphere; R, right hemisphere; Initial, initial MEG exam; 1 m, 1 month after initial exam (fixed stage); p, significance of differences between N100m source locations of two stages (x, y, and z coordinates, respectively); x: medial-lateral position relative to midline (positive=right); y: anterior-posterior position relative to the anterior commissure (positive=anterior); z: superior-inferior position relative to the commissural line (positive=superior). | | | | | | | | | | | | | | | | | | | | | | | |
