# Supplementary material for: Neuromagnetic Index of Hemispheric Asymmetry Prognosticating the Outcome of Sudden Hearing Loss
Source: PLoS One. 2012 Apr 20;7(4):e35055. doi: 10.1371/journal.pone.0035055 (PMC3332152; doi:10.1371/journal.pone.0035055)
Supplement: Table S3 — Amplitude and latency of peak dipole moment for N100m. (DOC) [file pone.0035055.s004.doc]

**Table S3. Amplitude and latency of peak dipole moment for N100m.**

| **Table S3. Amplitude and latency of peak dipole moment for N100m.** | | | | | | | | | | | | | | | | | | | |
| --- | --- | --- | --- | --- | --- | --- | --- | --- | --- | --- | --- | --- | --- | --- | --- | --- | --- | --- | --- |
|  | Control | | | | | |  | ISSNHL patient | | | | | | | | | | | |
|  |  |  |  | |  |  |  | Initial | | | | |  | | 1 m | | | | |
|  | Left | |  | | Right | |  | Healthy | |  | Affected | |  | | Healthy | |  | Affected | |
| Hemisphere | a | l |  | | a | l |  | a | l |  | a | l |  | | a | l |  | a | l |
| *Contralateral hemisphere* | | | | | |  |  |  |  |  |  |  |  | |  |  |  |  |  |
| 1 | 104.1 | 89.7 |  | | 52.1 | 95.3 |  | 55.3 | 80.5 |  | 65.9 | 86.0 |  | | 55.2 | 82.4 |  | 37.2 | 84.2 |
| 2 | 18.0 | 90.8 |  | | 30.6 | 98.9 |  | 20.7 | 76.8 |  | 37.4 | 111.9 |  | | 29.9 | 83.8 |  | 89.4 | 82.9 |
| 3 | 62.4 | 84.2 |  | | 55.1 | 91.6 |  | 35.5 | 117.4 |  | 113.5 | 98.9 |  | | 64.2 | 97.4 |  | 130.6 | 102.3 |
| 4 | 113.5 | 97.1 |  | | 49.9 | 106.3 |  | 66.2 | 82.4 |  | 16.8 | 138.1 |  | | 72.9 | 94.1 |  | 52.6 | 112.4 |
| 5 | 94.7 | 89.7 |  | | 90.6 | 99.5 |  | 39.7 | 128.5 |  | 50.3 | 110.1 |  | | 95.6 | 82.4 |  | 75.9 | 91.6 |
| 6 | 66.9 | 80.5 |  | | 55.0 | 81.7 |  | 87.2 | 84.1 |  | 54.8 | 114.5 |  | | 117.3 | 79.8 |  | 54.1 | 103.6 |
| 7 | 76.3 | 72.4 |  | | 48.1 | 76.1 |  | 38.7 | 78.7 |  | 89.5 | 100.8 |  | | 50.3 | 85.3 |  | 93.2 | 89.5 |
| 8 | 35.3 | 76.8 |  | | 59.4 | 87.9 |  | 31.8 | 74.3 |  | 60.0 | 100.0 |  | | 42.0 | 103.2 |  | 71.8 | 114.7 |
| 9 | 30.7 | 117.4 |  | | 41.7 | 98.9 |  | 54.5 | 75.9 |  | 35.3 | 121.2 |  | | 41.3 | 78.8 |  | 17.3 | 91.8 |
| 10 | 62.4 | 89.7 |  | | 55.1 | 121.1 |  | 44.2 | 76.1 |  | 29.2 | 142.2 |  | | 57.6 | 79.8 |  | 25.4 | 74.3 |
| 11 | 26.7 | 97.1 |  | | 46.8 | 78.5 |  | 50.3 | 72.3 |  | 76.1 | 84.6 |  | | 78.0 | 76.6 |  | 92.4 | 86.9 |
| 12 | 31.1 | 77.6 |  | | 37.1 | 111.0 |  | 51.9 | 93.9 |  | 69.5 | 110.0 |  | | 87.3 | 81.6 |  | 52.1 | 90.8 |
| 13 | 57.8 | 91.2 |  | | 46.7 | 87.7 |  | 48.4 | 78.8 |  | 35.3 | 78.8 |  | | 41.6 | 74.3 |  | 87.3 | 81.6 |
| 14 | 35.1 | 95.1 |  | | 37.3 | 78.9 |  | 82.3 | 81.6 |  | 114.1 | 74.3 |  | | 92.3 | 81.6 |  | 63.2 | 96.3 |
| 15 | 57.7 | 79.8 |  | | 40.7 | 85.3 |  | 63.8 | 81.6 |  | 76.8 | 82.0 |  | | 35.6 | 74.3 |  | 59.2 | 74.3 |
| 16 | 92.5 | 89.7 |  | | 66.5 | 97.1 |  | 80.1 | 81.6 |  | 93.8 | 83.4 |  | | 133.1 | 87.1 |  | 146.4 | 93.4 |
| 17 | 35.8 | 74.3 |  | | 63.5 | 81.6 |  | 30.6 | 87.1 |  | 76.6 | 74.3 |  | | 70.1 | 97.4 |  | 47.2 | 81.6 |
| 18 | 45.2 | 98.1 |  | | 52.1 | 98.1 |  | 28.0 | 98.1 |  | 10.4 | 156.8 |  | | 40.5 | 94.4 |  | 14.8 | 125.6 |
| 19 | 39.7 | 105.5 |  | | 65.5 | 105.5 |  | 33.7 | 88.9 |  | 63.3 | 72.4 |  | | 47.2 | 88.9 |  | 67.4 | 74.3 |
| 20 | 57.2 | 81.6 |  | | 42.6 | 81.6 |  | 36.4 | 74.3 |  | 96.1 | 76.3 |  | | 55.6 | 81.6 |  | 55.6 | 74.3 |
| 21 | 90.3 | 96.3 |  | | 59.7 | 88.9 |  | 37.6 | 81.6 |  | 42.3 | 88.9 |  | | 37.3 | 83.4 |  | 39.8 | 88.9 |
| m | 58.7 | 89.3 |  | | 52.2 | 92.9 |  | 48.4 | 85.5 |  | 62.2 | 100.3 |  | | 64.8 | 85.9 |  | 64.3 | 92.0 |
| SD | 27.7 | 10.9 |  | | 13.1 | 11.9 |  | 18.6 | 14.1 |  | 29.5 | 24.2 |  | | 28.6 | 8.2 |  | 35.6 | 14.6 |
| *Ipsilateral hemisphere* | | | |  | |  |  |  |  |  |  |  |  | |  |  |  |  |  |
| 1 | 47.9 | 108.2 |  | | 83.2 | 98.9 |  | 59.8 | 89.7 |  | 30.5 | 97.1 |  | | 46.1 | 89.7 |  | 55.5 | 91.6 |
| 2 | 15.4 | 87.9 |  | | 26.4 | 102.6 |  | 65.6 | 76.8 |  | 11.6 | 121.1 |  | | 85.7 | 89.0 |  | 35.6 | 75.8 |
| 3 | 26.2 | 104.5 |  | | 38.7 | 89.7 |  | 93.7 | 110.0 |  | 34.2 | 93.4 |  | | 110.2 | 97.4 |  | 56.4 | 102.2 |
| 4 | 37.2 | 122.9 |  | | 47.1 | 102.6 |  | 37.9 | 104.5 |  | 15.2 | 125.7 |  | | 58.5 | 109.7 |  | 59.6 | 112.4 |
| 5 | 36.8 | 106.3 |  | | 61.6 | 108.2 |  | 53.8 | 113.7 |  | 44.9 | 110.0 |  | | 72.3 | 90.2 |  | 74.7 | 100.8 |
| 6 | 51.6 | 91.6 |  | | 45.7 | 87.9 |  | 58.9 | 98.0 |  | 23.5 | 140.2 |  | | 94.4 | 83.4 |  | 46.2 | 129.3 |
| 7 | 16.6 | 100.0 |  | | 35.4 | 92.6 |  | 93.5 | 82.4 |  | 37.9 | 102.6 |  | | 100.9 | 88.9 |  | 26.2 | 89.5 |
| 8 | 38.1 | 100.8 |  | | 20.8 | 87.9 |  | 35.0 | 90.8 |  | 20.0 | 100.9 |  | | 35.5 | 108.0 |  | 65.6 | 107.8 |
| 9 | 33.0 | 106.3 |  | | 23.9 | 115.5 |  | 64.5 | 90.6 |  | 25.0 | 126.1 |  | | 31.5 | 130.2 |  | 48.8 | 117.9 |
| 10 | 26.2 | 98.9 |  | | 38.7 | 126.6 |  | 44.5 | 107.3 |  | 29.4 | 122.0 |  | | 26.8 | 83.4 |  | 22.6 | 103.6 |
| 11 | 39.8 | 101.6 |  | | 43.2 | 94.2 |  | 57.8 | 84.1 |  | 52.6 | 83.4 |  | | 51.4 | 76.6 |  | 78.8 | 86.9 |
| 12 | 26.8 | 112.9 |  | | 46.2 | 90.8 |  | 55.2 | 103.0 |  | 31.5 | 122.4 |  | | 73.4 | 96.3 |  | 38.8 | 88.9 |
| 13 | 30.6 | 100.1 |  | | 32.5 | 108.6 |  | 57.1 | 90.8 |  | 17.2 | 113.7 |  | | 42.7 | 88.9 |  | 29.6 | 90.8 |
| 14 | 20.8 | 104.1 |  | | 30.5 | 98.4 |  | 97.3 | 81.6 |  | 46.0 | 90.8 |  | | 110.0 | 81.6 |  | 54.3 | 105.5 |
| 15 | 29.1 | 92.6 |  | | 44.8 | 92.6 |  | 66.4 | 88.9 |  | 28.4 | 96.8 |  | | 111.5 | 81.6 |  | 30.6 | 88.9 |
| 16 | 43.1 | 106.3 |  | | 72.3 | 106.3 |  | 80.7 | 88.9 |  | 50.2 | 112.8 |  | | 117.0 | 94.4 |  | 98.7 | 90.8 |
| 17 | 19.4 | 105.5 |  | | 35.0 | 120.1 |  | 55.3 | 88.9 |  | 35.5 | 112.8 |  | | 44.7 | 97.3 |  | 35.6 | 105.5 |
| 18 | 43.3 | 105.5 |  | | 47.0 | 105.5 |  | 41.7 | 88.9 |  | 11.5 | 136.6 |  | | 46.5 | 92.6 |  | 18.6 | 142.2 |
| 19 | 37.3 | 112.8 |  | | 48.6 | 122.0 |  | 41.5 | 81.6 |  | 27.9 | 96.3 |  | | 51.6 | 81.6 |  | 55.3 | 96.3 |
| 20 | 35.7 | 96.3 |  | | 41.2 | 90.8 |  | 36.6 | 81.6 |  | 43.2 | 96.3 |  | | 36.3 | 88.9 |  | 21.6 | 96.3 |
| 21 | 34.7 | 105.5 |  | | 38.6 | 112.8 |  | 20.1 | 90.8 |  | 37.4 | 105.5 |  | | 34.2 | 87.1 |  | 16.6 | 112.8 |
| m | 32.8 | 103.4 |  | | 42.9 | 102.6 |  | 57.9 | 92.0 |  | 31.1 | 109.8 |  | | 65.8 | 92.2 |  | 46.2 | 101.7 |
| SD | 9.9 | 7.8 |  | | 15.0 | 11.8 |  | 20.4 | 10.3 |  | 12.1 | 15.5 |  | | 30.7 | 12.0 |  | 21.8 | 15.4 |
|  | a | l |  | | a | l |  | a | l |  | a | l |  | | a | l |  | a | l |
| *p_1_* | <0.001 | <0.001(Left*/Right* pooled) | | | | |  | 0.062 | 0.001(healthy*/affected* pooled) | | | | | 0.015 | | <0.001 |  |  |  |
| *p_2_* |  |  |  | |  |  |  | <0.001 | 0.677(healthy side dominance) | | | |  | | 0.142 | 0.441 |  |  |  |
| *p_3_* | <0.001 | <0.001 |  | | 0.002 | 0.002 |  | 0.023 | 0.020 |  | <0.001 | 0.024 |  | | 0.520 | 0.002 |  | 0.005 | 0.002 |
| Threshold for statistical significance using Wilcoxon signed rank test was set at P < 0.05. Left, left-ear stimulation; Right, right-ear stimulation; Healthy, healthy-ear stimulation; Affected, affected-ear stimulation; Initial, initial MEG exam; 1 m, 1 month after initial exam (fixed stage); a, amplitude of N100m dipole moment (Q/nAm); l, latency of N100m dipole moment (ms); m, mean; sd, standard deviation; P1, significance of difference between pooled responses of contralateral vs. ipsilateral hemispheres on monaural stimulation to both ears in controls and patients, respectively; P2, significance of difference between pooled responses of hemispheres ipsilateral to vs. opposite to the healthy ears on monaural stimulation to both intact and affected ears of patients; P3, significance of difference between hemispheric responses on a subset level according to ear of stimulation (left-ear stimulation and right-ear stimulation respectively in controls, healthy-ear stimulation and affected-ear stimulation respectively in patients). | | | | | | | | | | | | | | | | | | | |
